# Recurrent ST-Segment Elevation Myocardial Infarction (STEMI) in Localized Coronary Artery Ectasia Without Significant Obstructive Coronary Disease: A Therapeutic Dilemma

**DOI:** 10.7759/cureus.114414

**Published:** 2026-08-12

**Authors:** Emane Mengue Phalex, Laktib Nabil, Loubna El Khadir, Sylvie Ngomibe, Najat Mouine

**Affiliations:** 1 Cardiology, Mohammed V Military Teaching Hospital, Rabat, MAR; 2 Cardiology, Ibn Sina University Hospital, Rabat, MAR

**Keywords:** acute coronary syndrome, antithrombotic therapy, case report, coronary angiography, coronary artery ectasia, recurrent myocardial infarction, right coronary artery, st-segment elevation myocardial infarction

## Abstract

Coronary artery ectasia (CAE) is an uncommon angiographic abnormality characterized by the abnormal dilatation of the coronary arteries. Although frequently associated with atherosclerosis, CAE may promote sluggish coronary blood flow, thrombus formation, and distal embolization, resulting in acute coronary syndrome (ACS) even in the absence of significant obstructive coronary artery disease. The optimal management of ACS associated with CAE remains uncertain because current evidence is limited and no standardized therapeutic strategy has been established.

We report the case of a 52-year-old man with multiple cardiovascular risk factors and a previous inferior ST-segment elevation myocardial infarction (STEMI) who presented with recurrent inferior STEMI four years later. Emergency coronary angiography revealed marked localized ectasia of the proximal-to-mid right coronary artery measuring approximately 10 mm in diameter, containing a heterogeneous non-obstructive lesion (<30%) with preserved TIMI 3 flow and no angiographic evidence of acute vessel occlusion. Given the absence of a culprit lesion requiring revascularization, conservative medical management was adopted. The patient was treated with dual antiplatelet therapy, high-intensity statin therapy, beta-blocker, angiotensin-converting enzyme inhibitor, and therapeutic-dose low-molecular-weight heparin, with long-term oral anticoagulation planned because of recurrent thrombotic events.

This case highlights an uncommon mechanism of recurrent STEMI related to localized CAE without significant obstructive coronary disease. It emphasizes the diagnostic and therapeutic challenges posed by this condition and supports an individualized management strategy based on angiographic findings and thrombotic risk until stronger evidence becomes available.

## Introduction

Acute coronary syndrome (ACS) remains one of the leading causes of cardiovascular morbidity and mortality worldwide. Although the majority of ACS cases result from obstructive atherosclerotic coronary artery disease [1], approximately 5-10% of patients presenting with myocardial infarction have non-obstructive coronary arteries on angiography [2], representing a heterogeneous group with diverse underlying mechanisms.

Coronary artery ectasia (CAE) is an uncommon angiographic abnormality characterized by localized or diffuse dilatation of a coronary artery measuring at least 1.5 times the diameter of the adjacent normal coronary segment [3]. CAE is identified in approximately 0.3-5% of patients undergoing coronary angiography and is observed in up to 5% of patients presenting with acute myocardial infarction [4].

The management of ACS associated with CAE remains controversial. Percutaneous coronary intervention may be technically challenging because of the large vessel diameter, high thrombus burden, distal embolization, and risk of stent malapposition. In addition, the optimal antithrombotic strategy has not been established, and current guidelines provide no specific recommendations for these patients.

Because no standardized therapeutic strategy currently exists for ACS occurring in the setting of CAE, reporting additional cases may help improve clinical decision-making. We report a case of recurrent inferior ST-segment elevation myocardial infarction (STEMI) associated with localized right CAE without significant obstructive coronary artery disease, successfully managed with conservative medical therapy.

## Case presentation

A 52-year-old man presented with acute retrosternal chest pain suggestive of an ACS. His cardiovascular risk factors included well-controlled type 2 diabetes mellitus diagnosed in 2007, hypertension treated for four years, dyslipidemia under statin therapy since 2007, and a history of heavy smoking (60 pack-years), with smoking cessation four years earlier. A complete timeline of the patient's clinical course and major events is summarized in Table 1.

**Table 1:** Timeline of the patient's major clinical events STEMI: ST-segment elevation myocardial infarction; RCA: right coronary artery; ECG: electrocardiogram; LVEF: left ventricular ejection fraction

Relative day	Event
4 years before admission	First inferior STEMI managed conservatively. Coronary angiography demonstrated a non-obstructive thrombotic lesion of the proximal RCA
Day 0 (admission)	Onset of acute chest pain
Day 0 (+5h)	First medical evaluation. Twelve-lead ECG confirmed inferior STEMI. Dual antiplatelet therapy initiated
Day 0 (+10h)	Admission to our tertiary center. Persistent ST-segment elevation on ECG
Day 0	Transthoracic echocardiography: preserved LVEF (53%) with inferior wall hypokinesia
Day 0	Coronary angiography: localized RCA ectasia (~10 mm), non-obstructive thrombotic lesion (<30%), TIMI 3 flow
Day 1	Resolution of chest pain
Day 5	Hospital discharge with optimized medical therapy
Month 3	No recurrence of symptoms or thromboembolic events

**Figure 1:**
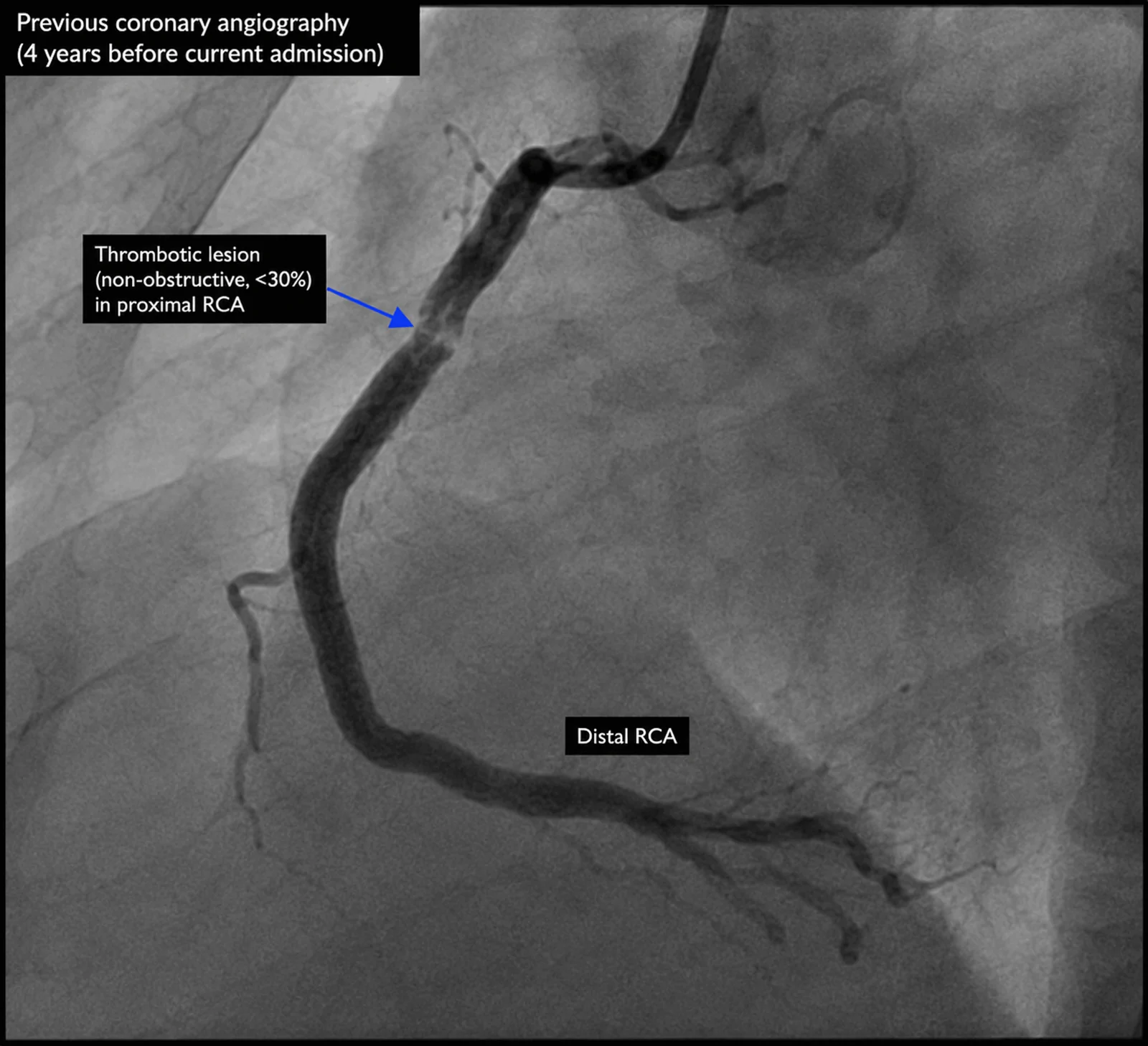
Previous coronary angiography performed four years before the current admission showing a non-obstructive thrombotic lesion of the proximal RCA (arrow) RCA: right coronary artery

Four years before the current admission, he had experienced an uncomplicated inferoposterior STEMI. Coronary angiography at that time demonstrated a non-obstructive thrombotic lesion of the proximal right coronary artery (Figure 1), and conservative medical treatment was preferred because no significant stenosis requiring revascularization was identified.

**Figure 2:**
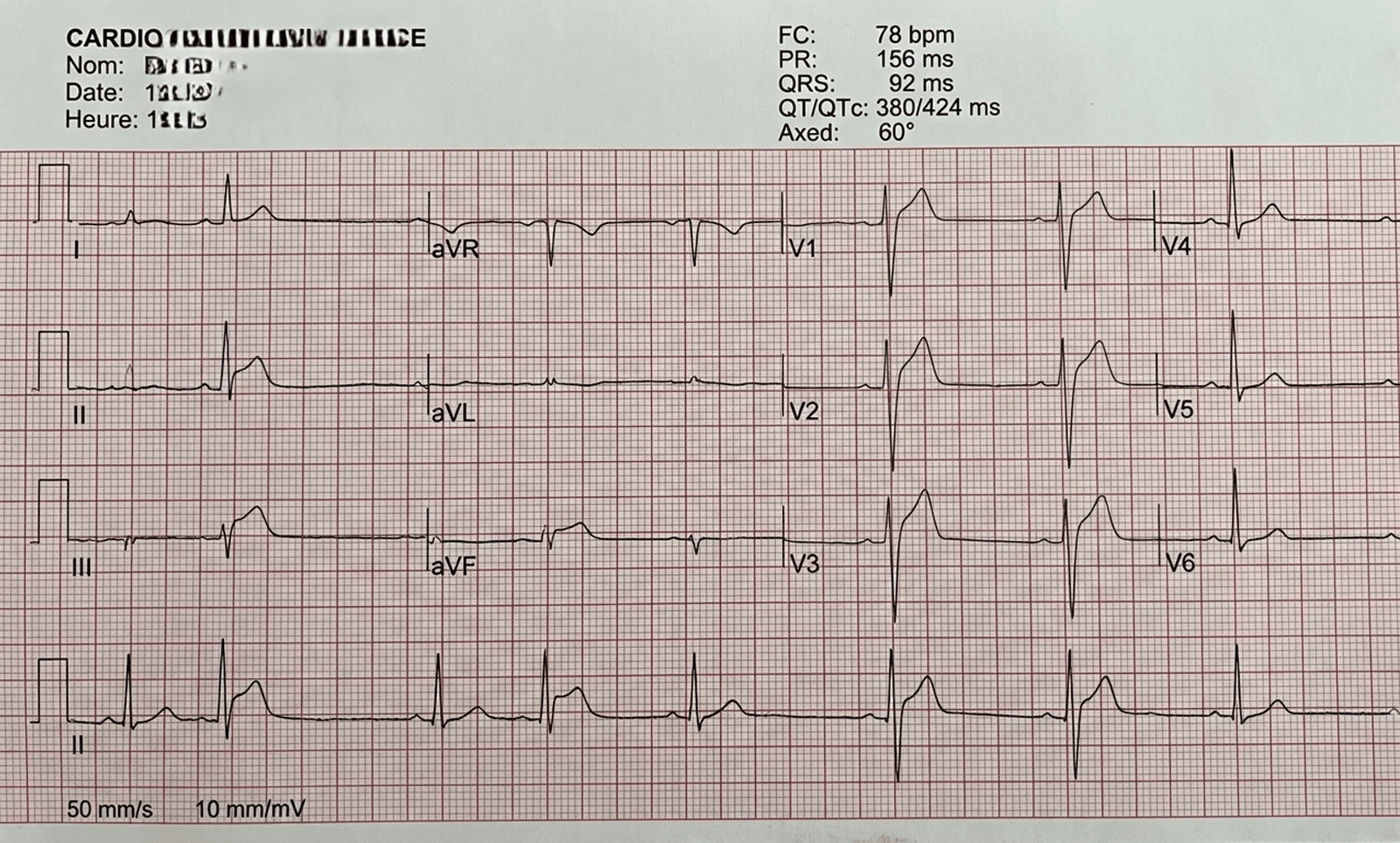
Initial 12-lead electrocardiogram obtained at the first medical contact demonstrating acute inferior ST-segment elevation myocardial infarction

The patient presented again with acute infarct-like chest pain. Five hours after symptom onset, he attended a private medical facility where a 12-lead electrocardiogram (ECG) confirmed an inferior STEMI (Figure 2) and dual antiplatelet therapy was initiated.

He was subsequently referred to our tertiary care center 10 hours after symptom onset for further evaluation and management.

**Figure 3:**
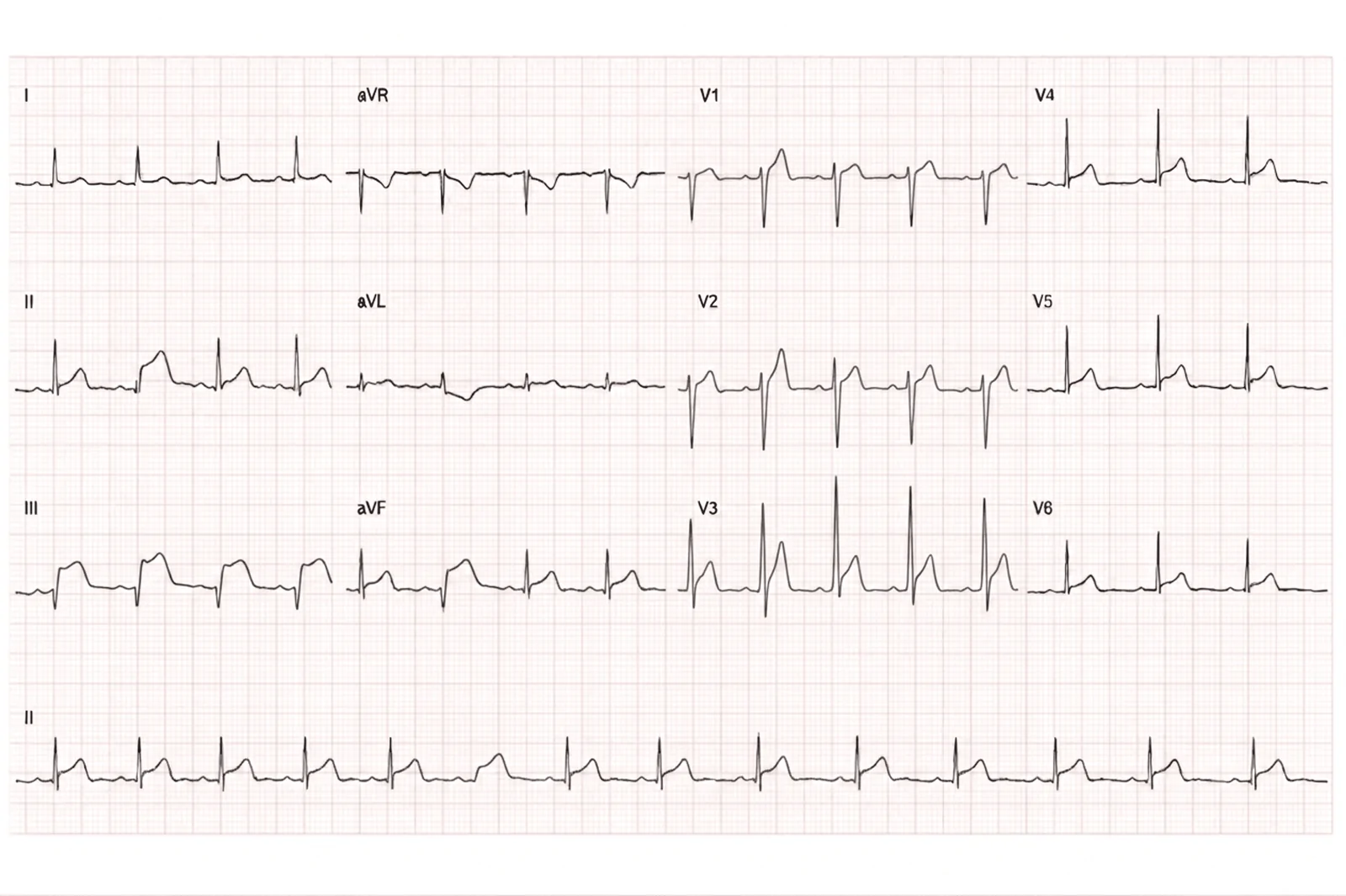
Admission 12-lead electrocardiogram demonstrating persistent ST-segment elevation associated with pathological Q waves in the inferior leads

On admission, the patient was hemodynamically stable and physical examination was unremarkable. The admission ECG showed persistent ST-segment elevation associated with pathological Q waves in the inferior leads (II, III, and aVF) (Figure 3).

**Figure 4:**
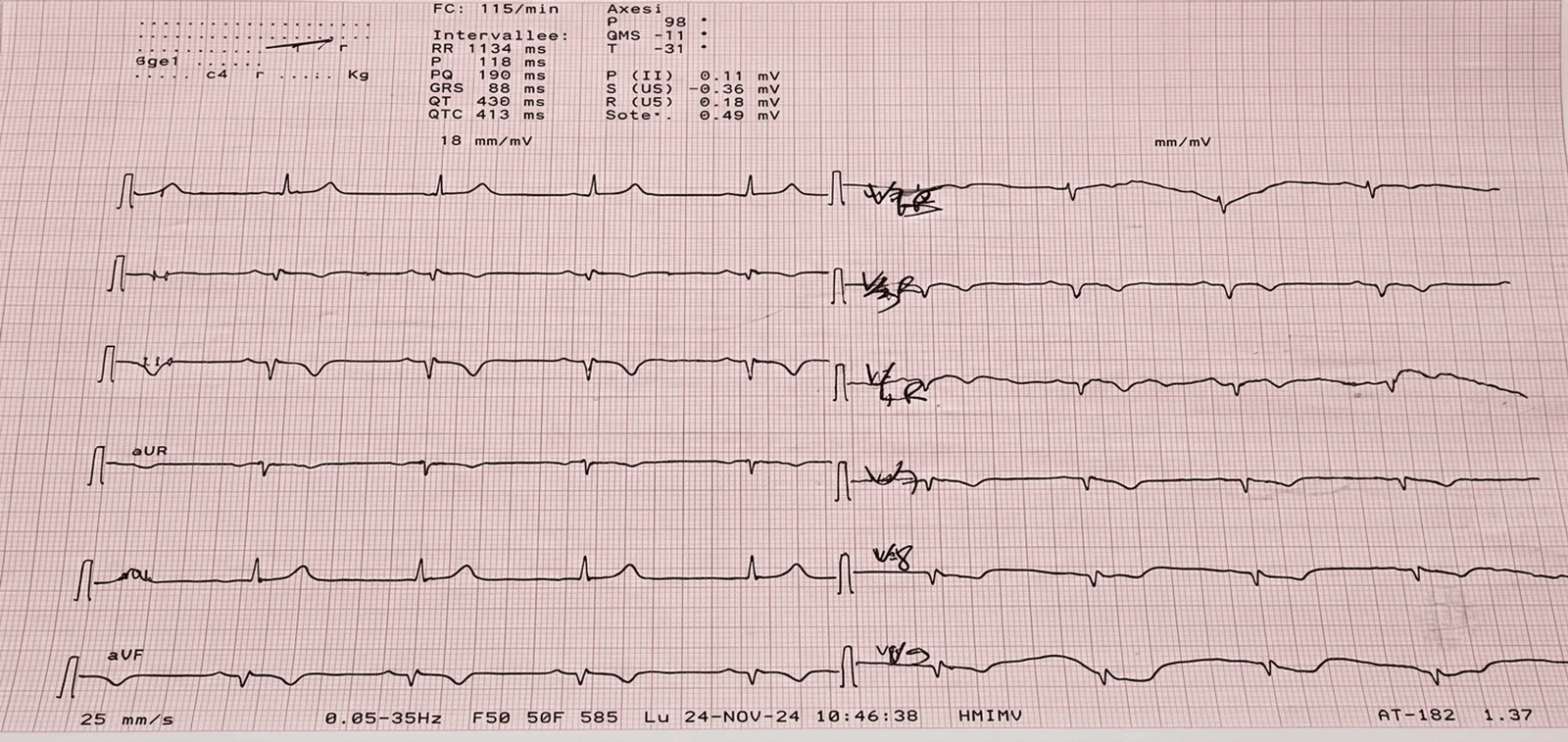
Posterior electrocardiogram leads showing pathological Q waves and T-wave inversion consistent with an inferoposterior myocardial infarction

Additional posterior leads demonstrated pathological Q waves with T-wave inversion in the posterior territory (V7-V9), consistent with inferoposterior myocardial infarction (Figure 4).

Chest radiography was normal. Transthoracic echocardiography revealed a non-dilated left ventricle with preserved global systolic function (left ventricular ejection fraction of 53%), regional wall-motion abnormalities consisting of inferior wall hypokinesia corresponding to the right coronary artery territory, and no significant valvular abnormalities. High-sensitivity cardiac troponin I was 3540 ng/L (252 times the upper reference limit).

**Figure 5:**
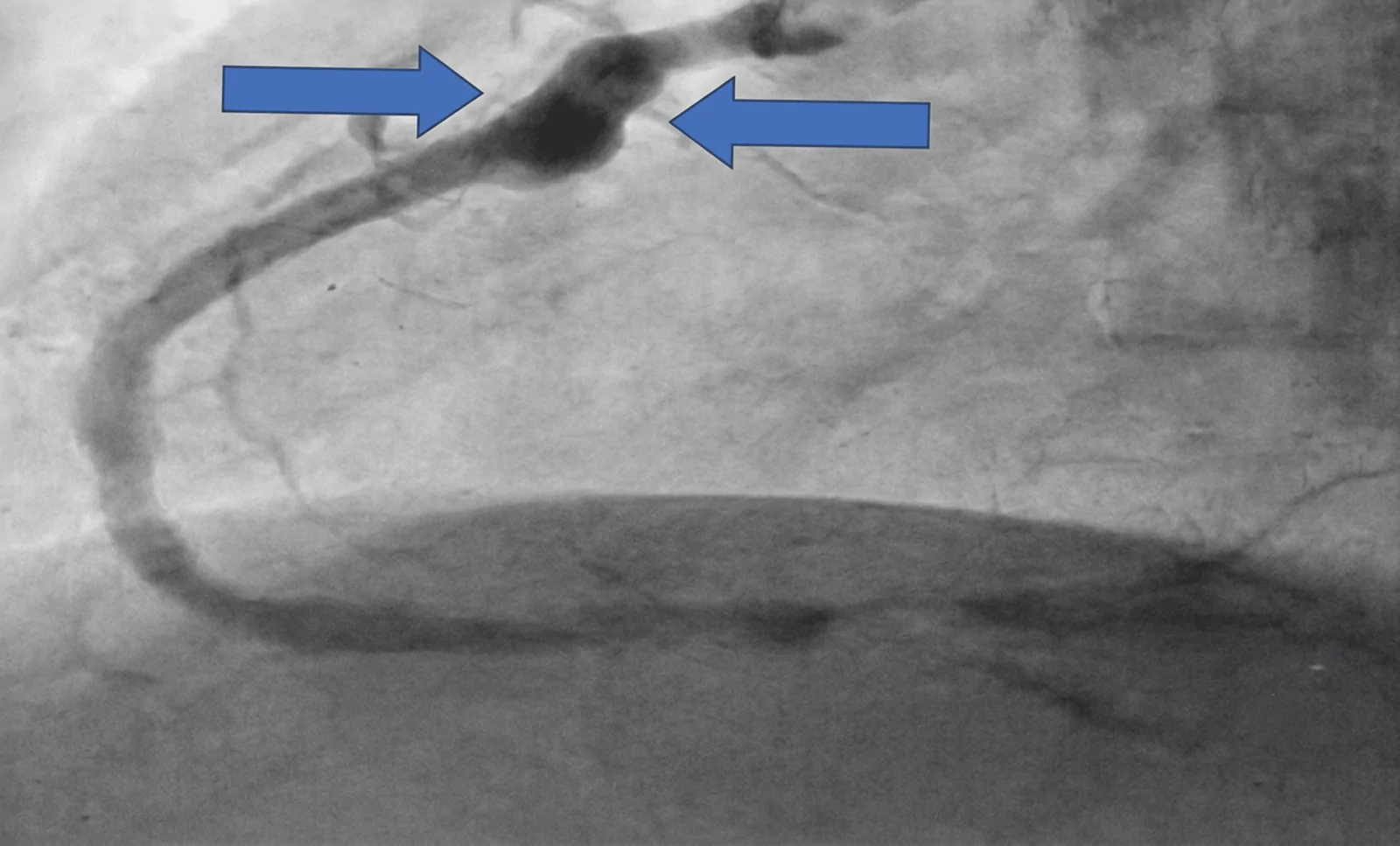
Coronary angiography demonstrating marked ectasia of the proximal-to-mid right coronary artery (blue arrows), measuring approximately 10 mm in diameter, with a heterogeneous non-obstructive lesion (<30%) and preserved TIMI 3 flow

Urgent coronary angiography demonstrated a markedly ectatic proximal right coronary artery measuring approximately 10 mm in diameter, with a heterogeneous non-obstructive lesion (<30%, American College of Cardiology/American Heart Association type B1 [5]), an abnormal distal vascular bed, and preserved TIMI 3 flow [6] (Figure 5). No angiographic evidence of significant stenosis, coronary dissection, plaque rupture, or coronary embolism was observed, and the left coronary system was angiographically normal.

The angiographic appearance was considered most compatible with a mural or organized thrombotic filling defect within the ectatic segment rather than a flow-limiting atherosclerotic stenosis. Nevertheless, plaque rupture, plaque erosion, or other intracoronary mechanisms could not be definitively excluded in the absence of intracoronary imaging. Corrected TIMI frame count and other quantitative coronary slow-flow indices were not routinely assessed during the angiographic procedure. Given the absence of an obstructive culprit lesion and the preserved distal coronary flow, percutaneous coronary intervention was not performed.

The patient was managed conservatively with optimized medical therapy, including dual antiplatelet therapy, high-intensity statin therapy, beta-blocker, angiotensin-converting enzyme inhibitor, and strict control of cardiovascular risk factors. Owing to the marked coronary ectasia and the presumed thrombotic mechanism underlying recurrent myocardial infarction despite non-obstructive coronary disease, long-term anticoagulation was discussed and individualized according to the patient's characteristics.

The patient's clinical course was favorable, with complete resolution of chest pain within 24 hours and no rhythm disturbances or signs of heart failure during hospitalization, and he was discharged on day 5 in a stable clinical condition. At the three-month follow-up, the patient remained asymptomatic, with no recurrence of chest pain or thromboembolic events.

## Discussion

CAE is an uncommon angiographic finding, first described by Björk [7] and later characterized by Markis et al. [8] as a distinct clinical entity. It is defined as a localized or diffuse dilatation of a coronary artery measuring at least 1.5 times the diameter of the adjacent normal coronary segment [8]. Its reported prevalence ranges from 0.3% to 5% among patients undergoing coronary angiography [9]. Although localized CAE and coronary artery aneurysm are often used interchangeably in the literature, some authors distinguish "aneurysms" as focal dilatations and "ectasia" as more diffuse arterial enlargement. In the present case, the angiographic appearance fulfilled the definition of localized CAE (Markis type IV), although a localized coronary artery aneurysm may also be considered according to alternative classifications.

Atherosclerosis is considered the most common etiology of CAE in adults [10,11], and the exact mechanisms leading to abnormal coronary dilatation remain incompletely understood. Histopathological studies suggest that excessive degradation of the extracellular matrix, mediated by increased metalloproteinase activity and reduced levels of their endogenous inhibitors, contributes to weakening of the vascular wall and progressive arterial dilatation [11,12]. Beyond structural arterial remodeling, increasing evidence suggests that the abnormal geometry of ectatic coronary arteries profoundly alters coronary hemodynamics. Enlargement of the vessel lumen results in disturbed wall shear stress, turbulent blood flow, prolonged blood residence time, and flow stagnation within the ectatic segment. These abnormalities promote endothelial dysfunction, platelet activation, local inflammatory responses, and activation of the coagulation cascade, thereby favoring mural thrombus formation and distal thromboembolism even in the absence of significant obstructive coronary disease.

Patients with CAE may remain asymptomatic or present with stable angina, ACS, or even sudden cardiac death [11]. In the present case, the patient experienced recurrent inferior STEMI four years after an initial myocardial infarction involving the same coronary territory. Interestingly, coronary angiography demonstrated a markedly ectatic proximal right coronary artery with only a non-obstructive (<30%) heterogeneous lesion and preserved TIMI 3 flow, supporting the hypothesis that coronary thrombosis and distal embolization rather than fixed obstructive atherosclerotic disease were the primary mechanisms responsible for recurrent myocardial infarction. However, because intracoronary imaging (intravascular ultrasound (IVUS) or optical coherence tomography (OCT)) and cardiac magnetic resonance imaging were not available, alternative mechanisms including plaque rupture, plaque erosion, spontaneous coronary artery dissection, coronary vasospasm, or coronary embolism cannot be definitively excluded. Consequently, the proposed ectasia-related thrombotic mechanism should be considered the most likely explanation rather than a definitive diagnosis.

According to the Markis classification [8], our patient had type IV coronary ectasia, characterized by the localized involvement of a single coronary vessel. Although this subtype is considered the least extensive form of CAE, it may still be associated with recurrent thrombotic events, as illustrated by our case. Although type IV ectasia is the least extensive form according to the Markis classification, localized ectatic segments may still constitute highly thrombogenic vascular territories because disturbed flow is largely determined by local vessel geometry rather than by the overall extent of ectatic disease.

The management of ACS associated with CAE remains challenging because no evidence-based therapeutic strategy has been established. In the present patient, conservative treatment was considered more appropriate than percutaneous coronary intervention because coronary angiography demonstrated preserved TIMI 3 flow without an obstructive culprit lesion requiring mechanical revascularization. Furthermore, the markedly enlarged vessel diameter would have increased the risk of incomplete stent apposition, persistent thrombus burden, distal embolization, and suboptimal procedural outcomes. Moreover, aspiration thrombectomy, intracoronary thrombolysis, covered stent implantation, and surgical revascularization have been reported only in selected cases of CAE complicated by extensive thrombus or giant coronary aneurysms. Consequently, treatment decisions should be individualized according to the angiographic findings and the patient's characteristics based on ischemic and bleeding risks [12].

Although the angiographic findings fulfilled the criterion of non-obstructive coronary artery disease, the identification of severe localized CAE provided a plausible ischemic substrate explaining the recurrent STEMI. Rather than representing a true myocardial infarction with non-obstructive coronary arteries (MINOCA), this case most likely reflected coronary thrombosis with distal embolization secondary to blood stasis within the ectatic coronary segment. Nevertheless, in the absence of intracoronary imaging or cardiac magnetic resonance imaging, the diagnosis should remain probabilistic rather than definitive.

The optimal antithrombotic regimen in CAE remains controversial [11,12]. Although current international guidelines provide no specific recommendations, several observational studies suggest that prolonged anticoagulation may reduce recurrent thrombotic events in selected high-risk patients, particularly those with extensive ectasia or recurrent ACS [13]. Nevertheless, the available evidence is almost exclusively derived from retrospective studies, observational cohorts, and expert opinion. To date, no randomized controlled trial has established the superiority of prolonged oral anticoagulation over antiplatelet therapy alone in patients with CAE. Consequently, decisions regarding long-term anticoagulation should be individualized after careful assessment of ischemic and bleeding risks.

Several observational studies have demonstrated that CAE is associated with adverse long-term cardiovascular outcomes [9,14]. Baman et al. reported increased mortality among patients with coronary artery aneurysms [10], whereas Wang et al. demonstrated poorer long-term outcomes in patients presenting with acute myocardial infarction and CAE [9]. More recently, Baldi et al. showed that STEMI patients with angiographic evidence of CAE had a significantly higher risk of recurrent myocardial infarction during follow-up, reinforcing the need for aggressive secondary prevention and close clinical surveillance [15]. Our patient's recurrent STEMI occurring four years after a previous infarction involving the same coronary territory further supports the concept that even localized coronary ectasia may be associated with recurrent thrombotic events despite the absence of severe angiographic stenosis.

This case highlights an uncommon mechanism of recurrent STEMI caused by localized coronary ectasia without significant obstructive coronary disease. It also illustrates the therapeutic dilemma encountered in these patients, in whom conservative medical treatment combined with intensified antithrombotic therapy may represent a reasonable individualized therapeutic option in carefully selected patients. However, conclusions regarding treatment effectiveness cannot be inferred from a single case because spontaneous clinical evolution and the effects of concomitant medical therapy cannot be separated.

This report has several limitations. First, intracoronary imaging (IVUS or OCT) and cardiac magnetic resonance imaging were unavailable, precluding the definitive characterization of the underlying mechanism of myocardial infarction and exclusion of plaque rupture, plaque erosion, spontaneous coronary artery dissection, or myocarditis. Second, corrected TIMI frame count and other quantitative indices of coronary slow flow were not assessed. Third, angiographic videos were unavailable for publication. Finally, as this is a single-case report with relatively short follow-up, no conclusions regarding treatment efficacy or the superiority of long-term anticoagulation can be drawn.

## Conclusions

This case highlights CAE as an uncommon but clinically important cause of recurrent STEMI in the absence of significant obstructive coronary artery disease. It emphasizes the prothrombotic nature of ectatic coronary arteries and the diagnostic and therapeutic challenges associated with this condition. In carefully selected patients with preserved coronary flow and no angiographic evidence of a culprit lesion requiring revascularization, an individualized conservative approach combining optimal medical therapy with tailored antithrombotic treatment may be considered.

However, given the absence of intracoronary imaging and the limited evidence supporting current management strategies, this report should be regarded as hypothesis-generating rather than evidence-establishing. Further prospective registries and randomized clinical trials are needed to define the optimal antithrombotic strategy, while broader use of intracoronary imaging and cardiac magnetic resonance imaging may improve diagnostic certainty and facilitate the development of standardized management algorithms for patients presenting with ACS associated with CAE.

## References

[1] Byrne RA, Rossello X, Coughlan JJ (2023). 2023 ESC guidelines for the management of acute coronary syndromes. Eur Heart J.

[2] Agewall S, Beltrame JF, Reynolds HR (2017). ESC working group position paper on myocardial infarction with non-obstructive coronary arteries. Eur Heart J.

[3] Bouzerda A, Bendriss L, Khatouri A (2018). Atheromatous coronary ectasia [Article in French]. Pan Afr Med J.

[4] Willner NA, Ehrenberg S, Musallam A, Roguin A (2020). Coronary artery ectasia: prevalence, angiographic characteristics and clinical outcome. Open Heart.

[5] Ryan TJ, Faxon DP, Gunnar RM (1988). Guidelines for percutaneous transluminal coronary angioplasty. A report of the American College of Cardiology/American Heart Association Task Force on Assessment of Diagnostic and Therapeutic Cardiovascular Procedures (Subcommittee on Percutaneous Transluminal Coronary Angioplasty). Circulation.

[6] (1985). The Thrombolysis in Myocardial Infarction (TIMI) trial - phase I findings. N Engl J Med.

[7] Björk L (1966). Ectasia of the coronary arteries. Radiology.

[8] Markis JE, Joffe CD, Cohn PF, Feen DJ, Herman MV, Gorlin R (1976). Clinical significance of coronary arterial ectasia. Am J Cardiol.

[9] Wang X, Montero-Cabezas JM, Mandurino-Mirizzi A (2021). Prevalence and long-term outcomes of patients with coronary artery ectasia presenting with acute myocardial infarction. Am J Cardiol.

[10] Baman TS, Cole JH, Devireddy CM, Sperling LS (2004). Risk factors and outcomes in patients with coronary artery aneurysms. Am J Cardiol.

[11] Dendramis G, Paleologo C, Lo Presti A (2014). Coronary artery ectasia: etiopathogenesis, diagnosis and treatment [Article in Italian]. G Ital Cardiol (Rome).

[12] Esposito L, Di Maio M, Silverio A (2021). Treatment and outcome of patients with coronary artery ectasia: current evidence and novel opportunities for an old dilemma. Front Cardiovasc Med.

[13] Grigorov V (2009). Invasive and anticoagulant treatment for coronary ectasia: a single operator's experience in a tertiary hospital in South Africa. Cardiovasc J Afr.

[14] Zhang Y, Huang QJ, Li XL (2015). Prognostic value of coronary artery stenoses, Markis class, and ectasia ratio in patients with coronary artery ectasia. Cardiology.

[15] Baldi C, Silverio A, Esposito L (2022). Clinical outcome of patients with ST-elevation myocardial infarction and angiographic evidence of coronary artery ectasia. Catheter Cardiovasc Interv.

